# Medical students who decompress during the M-1 year outperform those who fail and repeat it: A study of M-1 students at the University of Illinois College of Medicine at Urbana-Champaign 1988–2000

**DOI:** 10.1186/1472-6920-5-18

**Published:** 2005-05-19

**Authors:** Susan M Kies, Gregory G Freund

**Affiliations:** 1 College of Medicine, University of Illinois at Urbana-Champaign, Urbana, IL, USA

## Abstract

**Background:**

All medical schools must counsel poor-performing students, address their problems and assist them in developing into competent physicians. The objective of this study was to determine whether students with academic deficiencies in their M-1 year graduate more often, spend less time to complete the curriculum, and need fewer attempts at passing USMLE Step 1 and Step 2 by entering the Decompressed Program prior to failure of the M-1 year than those students who fail the M-1 year and then repeat it.

**Method:**

The authors reviewed the performance of M-1 students in the Decompressed Program and compared their outcomes to M-1 students who failed and fully repeated the M-1 year. To compare the groups upon admission, t-Tests comparing the Cognitive Index of students and MCAT scores from both groups were performed. Performance of the two groups after matriculation was also analyzed.

**Results:**

Decompressed students were 2.1 times more likely to graduate. Decompressed students were 2.5 times more likely to pass USMLE Step 1 on the first attempt than the repeat students. In addition, 46% of those in the decompressed group completed the program in five years compared to 18% of the repeat group.

**Conclusion:**

Medical students who decompress their M-1 year prior to M-1 year failure outperform those who fail their first year and then repeat it. These findings indicate the need for careful monitoring of M-1 student performance and early intervention and counseling of struggling students.

## Background

All medical schools are faced with poor performing M-1 students. The challenge is to encourage these students to take remedial programs that address their academic problems and assist them in becoming high performing physicians. During Academic Year 2001–2002, the LCME reported that 47 schools employed an Extended Time or Decompressed Program to assist poor performers [1]. Despite this widespread use of decompressed programs, little outcome information is reported in the literature.

In reviewing the literature, few outcome studies regarding remedial programs were found. Most articles discuss predictive measures of academic success in medical school, including both cognitive and non-cognitive variables that can assist admission committees to choose those students most likely to achieve well in medical school, while at the same time, steering committees away from admitting students likely to fail. Study results have provided medical school admission committees with data to apply to their admission policies and procedures [2-11]. Prominent among medical school admission policy and procedure changes are the development of 'prematriculation' programs and post baccalaureate programs, aimed at assisting the academically at risk students to find success after matriculation [12]. However, these policies and programs are not infallible and do not reflect the struggle failing students face, their rates of graduation, time spent in the program, number of attempts at USMLE Step 1 and Step 2. Importantly, curriculum track options were not discussed.

Once a student is admitted to medical school, it is important that academic progress be monitored. Typically, Student Affairs Offices and academic advisers are charged with this responsibility. Unfortunately, the literature has little information regarding the formulation and assessment of remedial programs [13]. Kassebaum and Szenas' study of all medical students matriculating from 1976 to 1988 revealed a decline in four year graduation rates, from 91.4% to 81.2%. During the same period, students requiring five years to graduate increased from 5.5% to 10.6%, with some students taking an additional sixth or seventh year to complete medical school [14]. The Alternative Curriculum developed at Boston University School of Medicine while not designed as a remedial program has become an entity where students experiencing academic difficulty enroll. McCahan [15] reported that half of those enrolled in the Alternative Curriculum either dropped out or were dismissed from medical school. Finally, in order to increase the retention of first year students at Indiana University School of Medicine, the Reduced Load Program was created in 1973. This remedial option allowed students to take two academic years to complete the first-year course requirements. In a study of this program, Ficklin, et al found it successful in retention of students, as 49% of this population, subject to dismissal for poor performance, graduated [16]. This research was conducted to provide students at risk for failure with the best possible guidance in choosing their curricular path.

At the University of Illinois College of Medicine in Urbana-Champaign approximately 125 M-1 students enroll each year. Since 1988, two options exist for M-1 students with performance deficiencies who wish to continue in medical school: the decompressed program or the repeat year. The decompressed program allows students to spread out their M-1 courses over two years. Students may opt into this program at any time between matriculation and one week after the M-1 second exam results are recorded. The repeat year is a full repeat of the entire curriculum. Repeat years are given to students, who have passed at least half of the M-1 curriculum after makeup examinations.

### Research question/hypothesis

We sought to determine the best remedial program for our students with academic problems in their first year of medical school. Based on working with students over several years, our hypothesis was: For failing M-1 students, those who entered the decompressed program would, as a group, perform better than those who repeated based on graduation rate, length of time to complete the curriculum, and the number of attempts at USMLE Step 1 and USMLE Step 2.

## Methods

Records of all medical students admitted to the M-1 track at the University of Illinois College of Medicine at Urbana-Champaign between 1988 and 2000 were examined (n = 1625). In this cohort, 107 students were found to have entered a remedial program to address M-1 deficiencies. The performance of these academically at risk M-1 students was analyzed comparing those in the decompressed program to those who fully repeated the M-1 year. After Institutional Review Board approval, 63 decompressed M-1 students were compared to 44 repeating M-1 students. Please note the students involved in the study are in the traditional curriculum. Medical Scholar Program Students (M.D./Ph.D.) were excluded from the study. Further, the cohorts are comparable as analysis was adjusted for differences in Cognitive Index and MCAT score. Subjects have comparable Cognitive Index scores and MCAT scores. 57% of the decompressed group students have underrepresented minority status while those in the repeating group 41% have underrepresented minority status.

Sample sizes of 63 in the decompressed group and 44 in repeating group are sufficient for having substantial power to detect differences between groups that are of practical importance. For instance, when using a two-sample t-statistic to test for a difference between the mean values of variables such as USMLE Step 1 scores, with a significance level of 0.05, these sample sizes result in 71% and 91% power, when the true means differ by 0.5 and 0.67 standard deviations, respectively.

To compare the decompressed and repeating groups, pre- and post- admission metrics were examined. The pre-admission metrics analyzed were: Cognitive Index (a proprietary formula that includes undergraduate GPA, rating of undergraduate institution and MCAT score); and MCAT score. The post-admission metrics analyzed were: graduation rates; length of time to graduation; and attempts to pass USMLE Step 1 and Step 2. For each comparison statistical analysis employed the Inman and Conover t Test (JMP Statistical Discovery Software, SAS Institute, Cary, NC). Possible confounding variables include potential personal pressures such as financial, health, and family issues. Any of these issues could have potentially affected subjects in this study, in both groups, but were unobtainable to the authors.

## Results

### Comparison of entering metrics

Predictive measures of success in medical school have been well studied [2-11]. However, dealing with medical students experiencing academic problems has not been well studied as outlined in the literature review above.

To compare our two groups upon admission, Inman and Conover t-Tests comparing the Cognitive Index of students and MCAT scores from both groups were performed. Results showed that there was no significant difference between the decompressed group and the repeaters relative to Cognitive Index (Table 1) or MCAT score (Table 2.).

**Table 1:** Comparison of Cognitive Index Between the Decompressed and Repeating Groups

Oneway Analysis of CI By Program t-Test						
	Difference	t-Test	DF	Prob>/t/		
Estimate	-0.0648	-0.063	101	0.9501		
Std Error	1.0331					
Lower 95%	-2.1141					
Upper 95%	1.9847					
Means and Standard Deviations Level	Number	Mean	Std Dev	Std Err Mean	Lower 95%	Upper 95%
Decompress	59	59.0586	5.34525	0.69589	57.666	60.452
Repeat	44	59.1234	4.9646	0.74849	57.614	60.633

This table shows there was no significant difference between the decompressed group and the repeating group relative to Cognitive Index scores. (Cognitive Index data not available for 4 students in the decompressed group.)

**Table 2:** Comparison of MCAT Between the Decompressed and Repeating Groups

Oneway Analysis of MCAT By Program t-Test						
	Difference	t-Test	DF	Prob>/t/		
Estimate	-0.38724	-1.411	102	0.1613		
Std Error	0.27448					
Lower 95%	-0.93167					
Upper 95%	0.15719					
Means and Standard Deviations Level	Number	Mean	Std Dev	Std Err Mean	Lower 95%	Upper 95%
Decompress	60	7.61253	1.33963	0.17295	7.2665	7.9586
Repeat	44	7.99977	1.4402	0.21712	7.5619	8.4376

Table Two shows there was no significant difference between decompressed group and the repeating group relative to MCAT score. (MCAT data not available for 3 students in the decompressed group.)

### Comparison of medical school performance

Table 3 shows that of the 63 students who enrolled in the decompressed program 58.7% (37 students) graduated compared to those 44 students in the repeat program where only 27.2% (12 students) graduated. Further, of those students in the decompressed program 46% (29 students) graduated in five years compared to the 12 students in the repeat program in which 18% (8 students), finished in five years. Of the decompressed group, 46% (29 students) passed USMLE Step 1 on the first attempt while 16% (7 students) of the repeating group passed on the first sitting of USMLE Step 1. Decompressed students passed USMLE Step 2 on the first attempt at the rate of 51% (n = 32), while Repeating students passed USMLE Step 2 on the first attempt at a rate of 21% (n = 9). Analysis of USMLE performance by groups also showed that there was a significant difference between groups on number of attempts at USMLE Step 1 (Table 4.) but no significant difference in the number of attempts on USMLE Step 2 (Table 5.).

**Table 3:** 1988–2000 Performance Data on Students in decompressed program and repeating program

	Decompressed		Repeated	
	Raw	%	Raw	%
Number Enrolled 88-00	63		44	
Number Graduated	37	58.7	12	27.2
For those who completed:				
Five Years to Graduate	29	46	8	18
Six Years to Graduate	4	6	3	7
Seven Years to Graduate	3	5	0	0
Eight Years to Graduate	1	2	0	0
Twelve Years to Graduate	0	0	1	2
1 Attempt to Pass Step 1	29	46	7	15.9
2 Attempts to Pass Step 1	7	11	1	2
3 Attempts to Pass Step 1	1	2	4	9
1 Attempt to Pass Step 2	32	50.8	9	20.5
2 Attempts to Pass Step 2	3	4.8	2	4.5
3 Attempts to Pass Step 2	2	3	1	2.3

Table Three contains Raw and Percentage data comparing students who either entered the decompressed program or repeated the M-1 year. Percentages are calculated using the number enrolled in each program during the M-1 year.

**Table 4:** Comparison of USMLE Step 1 Performance Between the Decompressed and Repeating Groups

Oneway Analysis of Step One By Program t-Test						
	Difference	t-Test	DF	Prob>/t/		
Estimate	-0.52616	-2.685	57	0.0095		
Std Error	0.19594					
Lower 95%	-0.91852					
Upper 95%	-0.13381					
Means and Standard Deviations Level	Number	Mean	Std Dev	Std Err Mean	Lower 95%	Upper 95%
Decompress	43	1.34884	0.612711	0.09344	1.1603	1.5374
Repeat	16	1.875	0.806226	0.20156	1.4454	2.3046

Table Four shows there was a significant difference between decompressed group and repeating group on the number of attempts at USMLE Step 1. (Note of the 63 students in the decompressed group, 43 students took USMLE Step 1, but 6 did not graduate. Of the 44 students in the repeating group, 16 took USMLE Step 1, of those 4 did not graduate.

**Table 5:** Comparison of USMLE Step 2 Between the Decompressed and Repeating Groups

Oneway Analysis of Step 2 By Program t-Test						
	Difference	t-Test	DF	Prob>/t/		
Estimate	-0.11712	-0.587	47	0.5598		
Std Error	0.19942					
Lower 95%	-0.5183					
Upper 95%	0.28407					
Means and Standard Deviations Level	Number	Mean	Std Dev	Std Err Mean	Lower 95%	Upper 95%
Decompress	37	1.21622	0.583816	0.09598	1.0216	1.4109
Repeat	12	1.33333	0.651339	0.18803	0.9195	1.7472

Table Five shows there was no significant difference on the number of attempts on USMLE Step 2 between the decompressed group and the repeating group.

## Discussion

These data demonstrate that upon admission to the College of Medicine at Urbana-Champaign student performance could not be predicted based on either MCAT score, nor on Cognitive Index score. Further, the selection of the decompressed program to aid poor performing M-1 students is the best predictor of success later in the medical school curriculum. Decompressed students graduated at a rate of 59% compared to repeating students who graduated at a rate of 27%. Decompressed students took less time to complete the curriculum and required fewer attempts at USMLE Step 1.

This research would be greatly enhanced if an additional comparison were made of those students who matriculated with the same admissions record data, but did not experience academic difficulty. This would allow for broader conclusions. Further, if the study analyzed additional data related to the relationship of variables such as financial stress, personal hardships, health etc., broader conclusions could be drawn. Unfortunately those data are unknown and unobtainable at this time.

## Conclusion

The results of this study demonstrate that the Decompressed Program is the best option for failing M-1 students, at the University of Illinois College of Medicine at Urbana-Champaign, as they performed better in the subsequent years of the curriculum. This study is a first step in understanding the remedial process with failing medical students. Further investigation is needed to develop criteria with which Student Affairs Offices and Promotions Committees can advise students on the most efficient/effective of handling M-1 remediation. In addition, identification of pre-enrollment metrics that would identify to medical school admissions committees those students likely to require participation in a decompressed program would be beneficial.

## List of abbreviations

Liaison Committee on Medical Education (LCME)

United States Medical Licensing Examination (USMLE)

## Competing interests

The author(s) declare that they have no competing interests.

## Authors' contributions

SK conceived of the study, designed the study, performed the statistical analysis and helped to draft the manuscript.

GF helped to refine the design of the study, has been involved in drafting the article and revising it critically for intellectual content.

Both authors read and approved the final manuscript.

## Funding

This research was supported by grants from the National Institutes of Health (DDK064862 to G.G.F.).

## Pre-publication history

The pre-publication history for this paper can be accessed here:


